# Structural and Functional Changes in the Na^+^/H^+^ Exchanger Isoform 1, Induced by Erk1/2 Phosphorylation

**DOI:** 10.3390/ijms20102378

**Published:** 2019-05-14

**Authors:** Larry Fliegel

**Affiliations:** Department of Biochemistry, University Alberta, Edmonton, AB T6G 2H7, Canada; lfliegel@ualberta.ca

**Keywords:** ERK (extracellular signal-regulated kinase), intrinsically disordered protein, Na^+^/H^+^ exchanger, pH regulation, phosphorylation, membrane transport, scaffolding

## Abstract

The human Na^+^/H^+^ exchanger isoform 1 (NHE1) is a plasma membrane transport protein that plays an important role in pH regulation in mammalian cells. Because of the generation of protons by intermediary metabolism as well as the negative membrane potential, protons accumulate within the cytosol. Extracellular signal-regulated kinase (ERK)-mediated regulation of NHE1 is important in several human pathologies including in the myocardium in heart disease, as well as in breast cancer as a trigger for growth and metastasis. NHE1 has a N-terminal, a 500 amino acid membrane domain, and a C-terminal 315 amino acid cytosolic domain. The C-terminal domain regulates the membrane domain and its effects on transport are modified by protein binding and phosphorylation. Here, we discuss the physiological regulation of NHE1 by ERK, with an emphasis on the critical effects on structure and function. ERK binds directly to the cytosolic domain at specific binding domains. ERK also phosphorylates NHE1 directly at multiple sites, which enhance NHE1 activity with subsequent downstream physiological effects. The NHE1 cytosolic regulatory tail possesses both ordered and disordered regions, and the disordered regions are stabilized by ERK-mediated phosphorylation at a phosphorylation motif. Overall, ERK pathway mediated phosphorylation modulates the NHE1 tail, and affects the activity, structure, and function of this membrane protein.

## 1. Introduction

**Figure 1:**
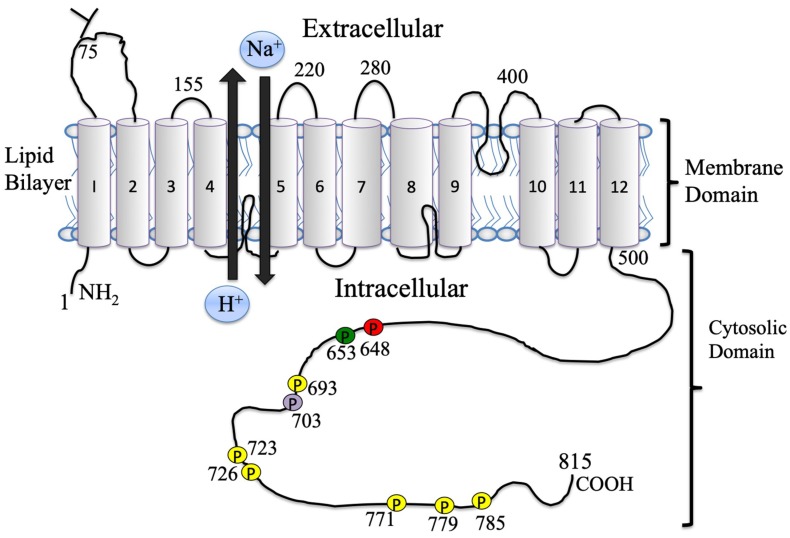
Illustration of the general structure of Na^+^/H^+^ exchanger isoform one, within the lipid bilayer. Transmembrane segments are labeled 1–12. Some representative amino acids on extramembrane loops are indicated. Amino acid 75 denotes the N-linked glycosylation site. The approximate position of phosphorylation sites is shown on the tail—yellow for extracellular signal-regulated kinase (ERK), red for protein kinase B (AKT), green for B-Raf, and purple for p90^rsk^; see Section 2 and Section 3 for details.

The mammalian Na^+^/H^+^ exchanger (NHE) is a ubiquitously expressed membrane protein that maintains intracellular pH (pH*_i_*), removing one intracellular H^+^ ion in exchange for a single extracellular Na^+^ ion [1]. NHE1 (isoform one) in the heart is implicated in both myocardial damage from ischemia/reperfusion injury and in promoting hypertrophy (reviewed in the works of [2,3]). This membrane protein consists of two domains. In humans, the 500 amino acid membrane domain transports ions and a 315 amino acid cytosolic domain regulates the membrane domain and is a target for protein interactions and phosphorylation [3,4] (Figure 1). Ten isoforms of NHEs exist; isoform 1 (NHE1) is the only plasma membrane isoform present in the heart and several other tissues [3,4,5,6,7,8,9].

### 1.1. NHE Structure and Subtypes Distribution

There are ten mammalian NHE isoforms (NHE1–10) that are products of different genes, with unique tissue distribution and physiological roles [4,9,10]. The first NHE cloned was NHE1 [11], and it is ubiquitously expressed in mammalian cells. NHE1 was identified by our laboratory as the predominant isoform in the myocardium [5,6], where it is concentrated along the intercalated discs and transverse tubule system, and is the only plasma membrane form of the protein present [12]. Many cell types lack NHE2–5 and 10 [13,14,15,16], while NHE6–9 are localized to intracellular organelle membranes such as mitochondria, endosomes, and the Golgi network [17,18]. NHE2 and NHE4 are mainly expressed in the gastrointestinal tracts, where NHE2 functions in Na^+^ reabsorption and may act with NHE4, promoting osmoregulation of renal inner medullary cells. NHE3 targets apical membranes of renal proximal tubule epithelia and the brush border membrane of mature intestinal epithelia, where plats an important role in Na^+^ reabsorption. NHE5 is expressed mainly in the brain and regulates pH in neurons. NHE6 is localized to mitochondria with high expression in high metabolic tissues such as the heart and brain (reviewed in the work of [10]). Intracellular Na^+^/H^+^ exchangers NHE6–9 may play a role in fine-tuning organellar pH and the cation composition of these intracellular compartments [17]. NHE10 is an osteoclast specific member of the family that regulates osteoclast differentiation [9].

### 1.2. NHE1 Physiological and Pathological Roles

NHE1 has a variety of roles in many cell types (see the works of [4,19,20,21] for reviews). Knockout of NHE1 from cells demonstrates its role in cell growth [22]. In a consanguineous human family, a deficiency in NHE1 caused ataxia and deafness [23], which was similar to NHE1-deficient mice [24,25]. NHE1 is also important in cell cycle progression [26,27] and in cell differentiation [28,29]. The tail of NHE1 anchors to the cytoskeleton via interactions with ERM (ezrin, radixin, moesin) proteins (which crosslink actin with the plasma membrane), affecting cytoskeletal structure, focal adhesion, and cell migration [30,31,32,33,34]. The role of NHE1 apoptosis varies with cell type. In mouse β-cells, trophic factor withdrawal triggers pH*_i_* dysregulation and apoptosis [35]. Activation of NHE1 leads to apoptosis in isolated cardiomyocytes [36]. NHE1 is involved in altering the pH*_i_* of malignant cells. NHE1-dependent alkalization plays a pivotal role in the development of a transformed phenotype [37,38,39,40]. NHE1 activation has been implicated as a key player in breast cancer cell invasion [41,42,43,44,45,46]. During ischemia, anaerobic glycolysis results in the production of protons, decreasing pH*_i_* and activating NHE1. Activated NHE1 exchanges internal H^+^ for extracellular Na^+^, leading to a rapid accumulation of Na^+^ in cells [47,48,49,50]. The high Na^+^ concentration drives an increase in Ca^2+^ via reversal of the Na^+^/Ca^2+^ exchanger. The resulting buildup of Ca^2+^ triggers various pathways leading to cell death. A huge body of evidence indicates that inhibition of NHE1 during ischemia and reperfusion protects the myocardium from this Ca^2+^ overload [47,48,49,50] (and see the works of [50,51] for reviews). NHE1 inhibition by the drugs cariporide, amiloride, and other benzoylguanidines is cardioprotective [52,53,54]. Activation of NHE1 regulatory pathways is important in NHE1-mediated damage to the myocardium [55]. Similarly, several studies have also shown that NHE1 inhibition prevents cardiac hypertrophy in vivo in rats [56,57] and mice [58,59,60,61,62,63,64,65].

### 1.3. The Na^+^/H^+^ Exchanger Structural Aspects

Transmembrane Na^+^/H^+^ exchange is ubiquitous across all phyla and kingdoms, so NHEs play an important role in many species. NHEs are grouped into the monovalent cation proton antiporter (CPA) superfamilies of CPA1, CPA2, and NaT-DC (Na-transporting carboxylic acid decarboxylase) [21]. The CPA1 family catalyzes Na^+^, Li^+^, K^+^, or Rb^+^ in the electroneutral exchange for a proton. CPA1 includes mammalian NHE1-9. The CPA2 family can catalyze electrogenic or electroneutral activity. This includes Na^+^, K^+^/H^+^ exchangers and the electrogenic *E. coli* NhaA antiporter. Additionally, it includes fungal antiporters and the mammalian electroneutral NHA1 and NHA2 proteins. NaT-DC transporters are a smaller group that export 1–2 Na^+^ in exchange for an extracellular H^+^ as part of a complex that catalyzes decarboxylation of oxaloacetate, malonyl/CoA, or glutaconyl/CoA [21].

The structures of four plasma membrane bacterial transporters Na^+^/H^+^ antiporters, *E. coli*, NhaA, [66], NapA of *Thermus thermophilus* [67], MjNhaP1 of *Methanocaldoccocus jannaschii* [68], and PaNhaP of *Pyrococcus abyssi* [69], have been elucidated by crystallography. The first known structure solved, *E. coli* NhaA, suggested that Na^+^/H^+^ antiporters have a novel fold. It consists of two transmembrane segments with a helix-extended region–helix conformation, which was TM4 and TM11 in the *E. coli* protein [70]. The *E. coli* protein also had a dimerization or scaffolding subdomain and a six-helix bundle cylindrical transport subdomain [66,71]. The NhaA fold was also found in TthNapA [67], MjNhaP1 [72], and PaNhaP [69]. EcNhaA is a dimer [73], as is MjNhaP1 [72]. Dutta et al. [70] recently published an alignment of Na^+^/H^+^ antiporters. The identity of various antiporters varied, being as low as 18% when comparing eukaryotic antiporters with *E. coli* NhaA. A yeast (*S. pombe*) Na^+^/H^+^ antiporter *Sp*NHE1 aligned reasonably with the 13 transmembrane segments of *Pa*NhaP and was predicted to have 13 transmembrane segments. Similarly, the plant Na^+^/H^+^ antiporter of Arabidopsis, SOS1, was aligned with a number of Na^+^/H^+^ antiporters and a 13 transmembrane segment topology was also predicted [74].

The topology of the *h*NHE1 isoform of the Na^+^/H^+^ exchanger is not yet deduced and is controversial. One model was made using cysteine-scanning accessibility and suggested a 12 transmembrane segment model with amino acids 15–36 N-terminal and cytosolic. [75]. Later, a 3D model was made using homology modeling with *Ec*NhaA [76]. Both models were similar except for different topology assignments of, and near, amino acids comprising TM9, 341–362. Later work suggested that amino acids 363–410 are EL5, with amino acids 341–362 preceding it as TM9 [77,78]. Recently, a newer molecular modeling of NHE1 also mapped amino acids 363–410 to the extracellular surface and also docked NHE inhibitors to sites on the protein [79]. The region between TM9 and TM10 (extracellular loop 5, approximately amino acids 362–411) was shown to be extracellular based on cysteine scanning and accessibility experiments. Part of this segment was suggested to be associated with the lipid bilayer based on its hydrophobicity and a lack of accessibility of a few residues in cysteine accessibility studies. However, the position of flanking amino acids confirmed it does not traverse the membrane [75,77]. It has since not been well studied.

The mammalian NHE isoforms share a greater identity within the membrane domain as opposed to the cytosolic regulatory domain. In the membrane domain, identity is conserved between transmembrane segments. This varies depending on the isoforms compared, but is often around 55, and rises to above 90% when comparing the same isoform in different species. In the hydrophilic C-terminal domain, the identity is approximately 24–31% [80]. It should be noted, however, that the structure of the cytosolic tail domain shows conservation (discussed below) [34].

## 2. Regulation of NHE1 Isoform of the Na^+^/H^+^ Exchanger, General

### 2.1. Rationale for Study of NHE1 Regulation

Regulation of the NHE1 isoform is extremely important. Not only is it important from the point of view of understanding fundamentally how the protein works, but also in human pathology. For example, it has been shown that artificially activating NHE1 activity by modulating regulation of the protein accentuates the damage the protein causes in pathology in the heart [59,81,82,83,84] and in breast cancer [46,85,86]—two common diseases. Alternatively, it has been suggested that targeting regulation of NHE1 warrants investigation to treat disease [87]. At the same time, while direct inhibition of NHE1 has been suggested to treat human disease, there have been detrimental off target side effects of NHE1 inhibitors in at least one clinical trial, though suppression of NHE1 activity still remains a potentially effective therapeutic approach to the treatment of human disease [88]. Clearly there is a need for a better understanding of NHE1 structure and regulation and application of this knowledge towards the treatment of human disease.

### 2.2. NHE1 Regulation

**Figure 2:**
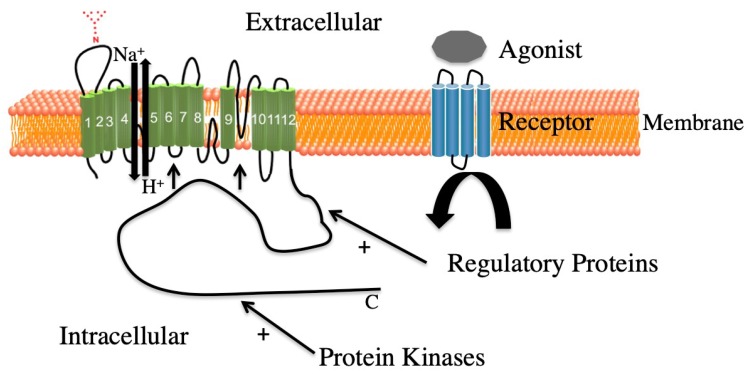
Regulation of Na^+^/H^+^ exchanger isoform 1 (NHE1). Through agonists, receptor mediated activation of the Na^+^/H^+^ exchanger, isoform 1, via protein regulatory interactions or through phosphorylation of the cytosolic C-terminus.

Various extracellular agonists mediate their effects through several cell surface receptors and signaling networks that modify the NHE1 C-terminal cytosolic regulatory domain. Modifications include binding of regulatory proteins that control transport activity by altering the affinity of the transport domain for intracellular H^+^ [10]. The particular agonists and coupling of receptors vary with the tissues involved. The pH dependence of NHE1 is usually shifted to a more alkaline range. Agonists include α_1_-adrenergic stimulation (phenylephrine) and hormones such as endothelin [89,90] (ET-1), thrombin, epidermal growth factor, angiotensin II, and lysophosphatidic acid [4,10,91,92,93,94,95,96,97]. This stimulation results in various binding proteins and protein kinases interacting directly and indirectly with NHE1 (Figure 2). An early study used deletion analysis and revealed that the distal 180 amino acids of the 315 amino acid C-terminal tail of NHE1 contain phosphorylation sites responsible for part of the growth factor induced activation of NHE1. Protein–protein interactions mediate other activation [98]. However, many of the protein binding sites and phosphorylation sites overlap [34]. Activation of NHE1 results in a shift of the pH sensitivity curve such that, at a given more alkaline pH*_i_*, the protein is more active.

Regulatory binding proteins were reviewed earlier [34]. Briefly, calmodulin is one of the most important of these and binds to two sites in the cytoplasmic tail of NHE1. One is a high affinity and one a low affinity site in amino acids 636–700 [99,100]. Calcium dependent binding to the high affinity site (636–656) blocks auto-inhibition, and thereby activates NHE1 [101]. A serine residue (Ser^648^) within the high affinity calmodulin binding site is phosphorylated by protein kinase B/Akt, reducing calmodulin binding [102]. Specific mutations to the auto-inhibition site can produce a hyper activated protein with significant physiological consequences [59,86].

Another calcium-regulated protein binding to the cytosolic regulatory domain is the calcineurin homologous protein group (CHPs). There are three CHP isoforms CHP1 [103], CHP2 [104], and CHP3 [105,106]. Like calmodulin, the CHP proteins bind calcium with high affinity and interact with NHE1 but at a different site, a juxtamembrane region of its tail domain at amino acids 516–540. CHP proteins may function to stabilize NHE1 in a physiologically optimal conformation, promoting protein stability and cell surface targeting [107].

Other proteins binding to the NHE1 tail include carbonic anhydrase II, which increases NHE1 activity and is dependent on the phosphorylation state of NHE1 [108]. The ERM family (ezrin, radixin, and moesin) forms links between actin filaments of the cytoskeleton and integral proteins of the plasma membrane [109], and NHE1 has ERM binding motifs in amino acids 552–560 of its cytoplasmic tail. Hsp70 and Hsp90 also bind to the C-terminus of NHE1 and may participate in protein folding [110,111,112,113].

Another regulatory binding cofactor is phosphatidylinositol 4, 5-bisphosphate. It binds to NHE1 in two juxtamembrane cationic binding regions, amino acid segments 513–520 and 556–564 (of the rat protein, equivalent to 509–516 and 552–560, respectively, of the human protein) [114]. Mutation of the sites did not prevent surface targeting but decreased efficiency of transport [114]. The interaction, induced by ATP depletion, inhibits NHE1 [114,115,116]. Aside from polyphosphoinositides, cationic regions of NHE1 also interact with acidic phospholipids including phosphatidylserine. Region 542–598 is thought to be a lipid interacting domain with the hydrophobic sequence ^573^LIAFY^577^ predicted to bind lipids [107,117]. Deletion or mutation of these residues results in decreased NHE1 activity.

### 2.3. NHE1 Regulation, Phosphorylation

#### 2.3.1. Phosphorylation, General

As noted above, phosphorylation of NHE1 is critical to the stimulation of activity and a number of different protein kinases have been shown to phosphorylate the cytosolic regulatory tail. Phosphorylation is suggested to account for about half of the growth factor induced regulation of NHE1 and is mostly thought to occur in the 180 amino acid distal region of the carboxyl terminal tail [4,118]. Earlier, we and others [3,4,34,44,119] reviewed phosphorylation-mediated regulation of NHE1 in several tissues. Briefly, some of the more well-characterized protein kinases that have been implicated in regulation through phosphorylation of the cytosolic domain are β-Raf (in the C-terminal 180 amino acids) [120], p38 Mitogen Activated Protein Kinase (MAPK) (Thr^718^, Ser^723^, Ser^726^, Ser^729^, rabbit sequence changed to equivalent human) [35], and protein kinase B/Akt (Ser^648^, [102,121] (a more extensive list includes MS-based phosphoproteomics and is found in the work of [34]).

#### 2.3.2. ERK Mediated Regulation, General

**Figure 3:**
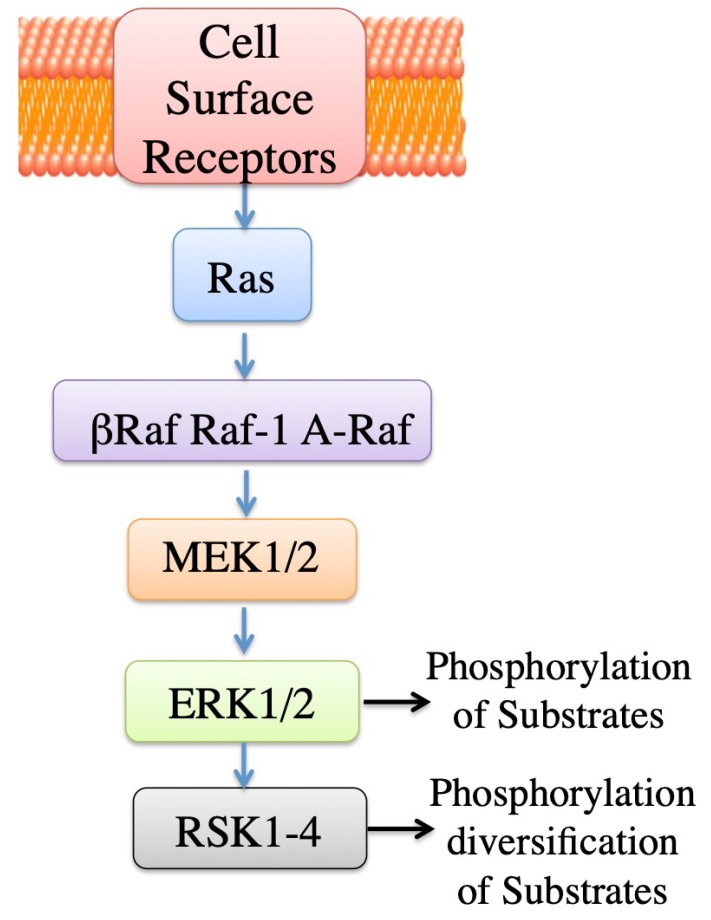
Simplified schematic of the ERK signaling cascade. Activation processes are indicated. Raf family members bind Ras/GTP (not shown). Raf isoforms activate ERK kinase (MEK). Activated ERK has many substrates. Downstream of ERK1/2 is the RSK family with various isoforms, which participate in ERK signaling and diversification of the signal. Adapted from the works of [122,123].

The ERK cascade is a central pathway transmitting signals from many extracellular agents such as hormones to regulate a variety of processes including proliferation and differentiation. Signaling via this cascade pathway travels through sequential phosphorylation involving activation of protein kinases in different levels of the cascade. Briefly described, the main components of the chain of phosphorylation are the Raf kinases, MEK1/1, ERK1/2 (the ERKS), and ribosomal protein S6 kinase (RSKs) (Figure 3). There are also other components including spliced forms and different proteins that participate depending on conditions, for details see the works of [122,123].

The ERK pathway is important in many aspects of human health and pathology with a number of similarities to the role of NHE1. ERK1/2 are important in cell growth and differentiation [123], as is NHE1 [4]. Mutations in the ERK pathway are important in triggering cancer [123] and activated NHE1 is important in cell growth and has been implicated as a trigger in several kinds of cancers including ovarian, breast, and prostate cancer [44,120,124,125,126]. Similarly, the ERK pathway is involved in several aspects of heart disease including cardiac hypertrophy and ischemia reperfusion damage to the heart [127,128], and NHE1 is involved in the aggravation of both of these forms of myocardial disease [3,107]. The disease states of ischemia and hypertrophy in the myocardium and metastatic breast cancer are known to have activated ERK pathways that activate the Na^+^/H^+^ exchanger [3,86,129]. Below, we describe evidence that demonstrates that ERK directly phosphorylates and regulates NHE1 in two disease states.

## 3. ERK Mediated Regulation of NHE1

### 3.1. ERK Pathway Regulation of NHE1 in the Myocardium

Early studies showed that ERK-dependent phosphorylation of NHE1 was hormonally regulated and occurred in the cytosolic domain [130]. Later, it was demonstrated that ischemia and reperfusion of the mammalian heart activated ERK1/2 and increased their activity towards the Na^+^/H^+^ exchanger. Both ERK1/2 and p90^RSK^ activity towards NHE1 was increased [55]. Ischemia causes intracellular acidosis, which activates the ERK1/2 pathway. It was thus also demonstrated that artificially inducing acidosis in isolated cardiomyocytes activates the ERK1/2 pathway and increases Na^+^/H^+^ exchanger activity. This was through the Ras/Raf/MEK pathway [131]. Another form of activation is through bursts in reactive oxygen species that occur in cardiac ischemia reperfusion. One of these, H(2)O(2), has also been shown to activate ERK1/2 and increase phosphorylation and activation of the Na^+^/H^+^ exchanger in cardiomyocytes [132,133,134].

The sites of phosphorylation of NHE1 in this pathway have obviously attracted interest. Some of the first studies showed that Ser^703^ is directly phosphorylated by p90 ribosomal S6 kinase (p90^RSK^), causing activation of NHE1. Additionally, phosphorylation of this site (RIGSDP) causes association of the protein 14-3-3 and activation of NHE1 [135]. It is notable that inhibiting p90^RSK^-mediated phosphorylation of this site reduces myocardial infarction caused by coronary artery occlusion [136]. Clearly, phosphorylation at this site plays an important physiological role in the heart, a point that has been verified pharmacologically [128].

**Figure 4:**
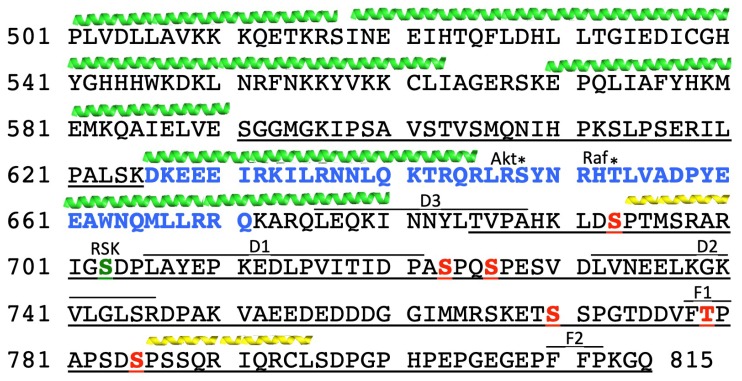
Amino acids of the cytosolic regulatory C-terminal domain of NHE1, with features indicated. Intrinsically disordered amino acids are underlined [34]. Amino acids phosphorylated by ERK and identified in the work of [142] are shown in red. Calmodulin binding domain, blue/bold. Ser^703^ phosphorylated by p90^RSK^, bold and green. Thr^653^, phosphorylation site of β-Raf, indicated by *. Ser^648^, AKT phosphorylation site, indicated by an asterisk. D-domain and F-site putative ERK binding sites [142] are indicated with a line over the amino acid. Green helices above amino acids are the approximate location of four predicted helices, according to the authors of [34]. Yellow helices above amino acids indicate transient helices induced by ERK-mediated phosphorylation, according to the authors of [145].

While Ser^703^ plays an important role in the myocardium, it is not the only site phosphorylated via the ERK dependent pathway. Two groups studied this. We [137,138,139,140] identified direct phosphorylation of the NHE1 cytosolic domain by ERK1/2. This was demonstrated in four general regions, 1, Ser^693^; 2, Thr^718^,Ser^723/726/729^; 3, Ser^766/770/771^; and 4, Thr^779^,Ser^785^, which were identified by mass spectrometry [3,139] (Figure 4). ERK-dependent phosphorylation of Ser^770^ and Ser^771^ was demonstrated in rat cardiomyocytes and Chinese hamster ovary (CHO) cells in response to hormonal stimulation and sustained acidosis [137,140]. ERK-dependent sustained intracellular acidosis was shown to activate the NHE1 protein in CHO cells and in cardiomyocytes, even when Ser^703^ had been mutated to Ala, demonstrating that the ERK-dependent pathway can activate NHE1 without p90^RSK^ and Ser^703^ phosphorylation [141]. A second group [142] similarly demonstrated phosphorylation of the NHE1 tail by ERK2. The six sites Ser^693^, Ser^723^, Ser^726^, Ser^771^ and Thr^779^, and Ser^785^ were phosphorylated in a distinct temporal order with varying rate constants. Phosphorylation of residues Ser^693^ and Thr^779^ occurred first and simultaneously, and other phosphorylation appeared later (Figure 1 and Figure 4).

An indication that phosphorylation results in structural changes in the cytosolic regulatory domain was first suggested in 2013 [143]. By making phosphomimetic mutations, it was demonstrated that the full-length protein had altered accessibility to trypsin digestion. The same phenomenon occurred in a peptide fragment. Hydrogen/deuterium exchange mass spectrometry showed that the phosphomimetic changes (S770D, S771D) appear to stabilize a sequence between residues Met^764^ and Thr^779^. Conversely, the region between amino acids Tyr^577^–Leu^588^ was less stable in the phosphomimetic protein [143].

Another member of the ERK pathway, β-Raf, has also been shown to phosphorylate the C-terminal domain of the Na^+^/H^+^ exchanger and activate the protein. Originally, it was demonstrated that in patients with the V600E mutation of β-Raf, there is aberrant pH regulation in malignant melanoma cells due to stimulation of NHE1 [120,144]. Later, it was shown that in cardiomyocytes, inhibition or depletion of β-Raf inhibited NHE1 activity. Additionally, it was demonstrated that β-Raf bound to NHE1 and phosphorylated amino acid Thr^653^ (Figure 1 and Figure 4) [120].

### 3.2. ERK Pathway Regulation of Breast Cancer Metastasis

NHE1 is a trigger for metastasis in triple negative breast cancer cells [44,85,124,146,147,148,149,150,151,152]. It facilitates metastasis in part, by acidifying the extracellular matrix region promoting activity of extracellular digestive enzymes [37,44,148,153,154,155]. The extracellular acidification is thought to be necessary for protease activation, which facilitates the digestion and remodeling of the extracellular matrix [149,153,155], critical in metastasis. Elevated NHE1 activity occurs in triple negative breast cancer cells, which facilitates the growth and metastasis of triple negative breast cancer cells [85]. Inhibition or knockout of NHE1 can reduce growth and metastasis of triple negative breast cancer cells [85,86]. Loss or inhibition of NHE1 increases the susceptibility of breast cancer cells to paclitaxel in triple negative breast cancer cells [85].

A key regulatory site of NHE1 in breast cancer is Ser^703^, which is phosphorylated by p90^RSK^ (Figure 1 and Figure 4). Mutation of this p90^RSK^ phosphorylation site on NHE1’s cytosolic C-terminal domain, to the non-phosphorylatable Ala, alters the morphology of invasive mesenchymal-like MDA-MB-231 cells to the less invasive, smaller, rounder, and non-invasive epithelial phenotype. There is also a loss of expression of the protein and mRNA of the mesenchymal marker vimentin [86]. Affinity chromatography and co-immunoprecipitation confirmed an interaction between NHE1 and 14-3-3 [156], which binds to this site. Pharmacological inhibition of p90^RSK^ also inhibited the metastatic potential of invasive MDA-MB-231 cells [86]. Thus, in this disease model, the ERK pathway and certainly Ser^703^ play an important role in the pathology of the disease. It has also been shown that expression of a truncated, constitutively active ErbB2 tyrosine kinase receptor in MCF-7 breast cancer cells leads to increased ERK1/2 and p90^RSK^ activity. This mutant protein is associated with increased metastatic potential and cell motility and caused increased phosphorylation of Ser^703^ of NHE1 [157]. It is also worth mentioning that an association of elevated NHE1 expression with p90^RSK^ has also been shown in primary metastatic basal triple negative breast cancer tumors [156].

## 4. NHE1 Scaffolding, ERK1/2, and Disordered Domains

### 4.1. Scaffolding and ERK1/2

As noted above, the tail of NHE1 has several interacting proteins. The regulatory tail of NHE1 may assemble signaling complexes in special plasma membrane domains, in order to coordinate signaling pathways and complexes [19,158] that promote signaling, or regulatory events and complex formation [159]. Meima [19] postulates that the NHE1 scaffolding ensemble is not static, but varies in response to different stimuli. This has not yet been well documented empirically. The NHE1 “interactome” was examined by affinity chromatography and immunoprecipitation in MDA-MB-231 breast cancer cells. NHE1 was associated with 14-3-3 protein, AKT kinase, alpha-enolase, CHP1, and heat shock proteins HSP70 and HSP90 [156]. A similar approach with renal tissue also demonstrated the presence of interacting heat shock proteins and 14-3-3 [111]. One group [158] used siRNA knockdowns and yeast two-hybrid screening to demonstrate that NHE1 is a MAPK scaffold for several members of the MAPK family, including ERK and Raf [158]. More recently, another group [142] identified the NHE1 tail as a scaffold for ERK1/2. These S/T kinases interact with targets and regulators via two kinds of domains, D-domains and F-sites. D-domains (also known as docking sites for ERK) have the sequence of 2–5 basic residues (R/K), which is spaced by 1 to 6 residues, followed by a motif hydrophobic amino acid -X-hydrophobic amino acid (Φ-X-Φ, the hydrophobic is usually V, L, or I) (Figure 4). F-sites, also called DEF (docking site for ERK, FXFP)-domains, have the canonical FXFP sequence. They allow for aromatic residues at the P1 (F, W) and P3 positions (F, Y, W). NHE1 has three D-domains and two F-sites (Figure 4) identified in its tail sequence. Their results showed that ERK2 interacted with the D3 domain and two F-sites [142]. Mutation of the D-domains from Φ-X-Φ to AXA showed that only the D3 domain was important for ERK interactions in vitro and in CHO (Chinese hamster ovary) cells. Mutation of the F sites from FTP778–780 to ATP778–780 and FP811–812 to AA811–812 both affected ERK interactions in vitro and mutation of the F1 site affected interactions in CHO cells (F2 was not tested). Thus, the D3 domain and two F-sites are ERK interaction sites.

### 4.2. ERK1/2 Scaffolding and the Disordered Domains of NHE1

One interesting structural aspect of the Na^+^/H^+^ exchanger family is found in the cytosolic regulatory, 315 amino acid tail. This region is poorly conserved among isoforms. This probably lends specificity to the regulation of the isoforms. The proximal part of the NHE1 tail is ordered and four alpha helices are predicted from amino acids 506 to 590. However, this is followed by a predicted disordered region (amino acids 591–625). There are two helices predicted between amino acids 626–685, but the distal part of the tail from amino acids 686–815 is predicted to be intrinsically disordered (Figure 4). This character is common and conserved in the NHE tails of various types [34]. Intrinsic disorder often results from charged amino acids and from their low hydrophobicity, which leads to increased flexibility. This flexibility leads to a variety of interchangeable conformations that are key to binding interactions that assist in molecular recognition. In many cases, there may be linear short binding motifs within the flexible regions and these intrinsically disordered proteins may fold when other proteins bind [34,160,161].

### 4.3. ERK1/2 Phosphorylation and Binding and Conformational Changes in the NHE1 C-terminus

As noted above, an early study [143] demonstrated that phosphomimetic mutations in the cytosolic C-terminus of NHE1 caused conformation changes in the tail. A recent and elegant study [145] examined more directly the effect of phosphorylation of the C-terminal of NHE1 via ERK2 using nuclear magnetic resonance (NMR) spectroscopy. They examined the ERK2 phosphorylation sites Ser^693^, Ser^723^, Ser^726^, Ser^771^, Thr^779^, and Ser^785^, as well as changes in conformation using NMR chemical shifts. A secondary structure analysis by NMR revealed four transient helices between amino acids 694 and 795 in the disordered regions of the tail domain. The ERK2 phosphorylation sites were located N-terminal to transient helices 1–4 at 694–700, 727–743, 758–766, and 786–795. With phosphorylation of the six ERK2 sites, the local transient helices 1 and 4 showed increased helicity. Arg^790^ was important for helix stabilization upon Ser^785^ phosphorylation, while an upstream acid residue (Asp^784^) was not. The motif [S/T]-P-{3}-[R/K] was suggested to be a helix promoting motif with the Pro as a possible starting point for the helix.

## 5. Conclusions

### 5.1. Summary of ERK Pathway Mediated Effects

**Figure 5:**
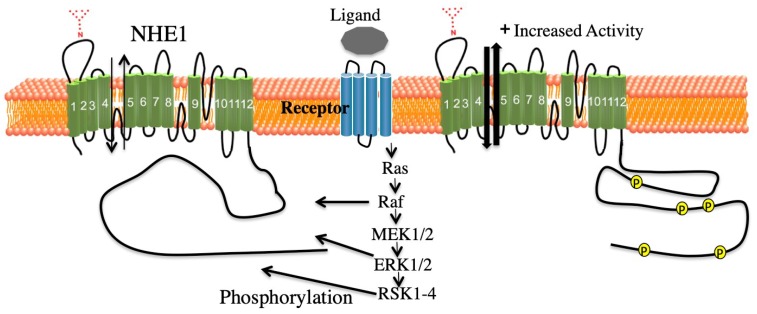
Simplified schematic of the hypothetical ERK signaling cascade that activates the Na^+^/H^+^ exchanger. Cell surface receptor activates ERK signaling cascade that causes multiple kinases, including ERK, to phosphorylate the NHE1 regulatory cytosolic domain. ERK1/2 phosphorylates in multiple locations changing the conformation of the cytosolic domain, activating the membrane domain. Downstream of ERK1/2 is the RSK family that participates in ERK signaling and NHE1 phosphorylation, and upstream is the Raf family that also participates in NHE1 phosphorylation.

It is clear that there are a number of regulatory mechanisms and physiological effects of regulation of NHE1 in several tissues. Perhaps the most significant to mankind is the role the protein plays in heart disease and breast cancer, which affect relatively large percentages of the whole population and of women, respectively. Elevated activity of the NHE1 isoform of the Na^+^/H^+^ exchanger is a trigger for damage in ischemic heart disease and is an important trigger for cell growth and metastasis in breast cancer. The cytosolic C-terminal tail of NHE1 serves as a regulator of transport of the membrane domain, but more than that, it is a focal point for regulation of the protein that occurs principally by protein binding and phosphorylation (and lipid binding). ERK mediated phosphorylation of the C-terminal occurs at multiple sites and affects the structure and function of the protein. The regulatory tail itself possesses both structured and unstructured intrinsically disordered domains. The intrinsically disordered domains are important in the function of the protein and phosphorylation by ERK affects their structure, inducing helical structure in regions adjacent to the phosphorylation site. We suggest that this conformational change in the tail affects the membrane domain, altering its affinity for protons and increasing activity at more alkaline intracellular pH (Figure 5).

### 5.2. Future Studies

The intrinsically disordered regions of the cytosolic domain of the Na^+^/H^+^ exchanger are of great interest. Clearly, they are involved in the regulation of the NHE1 protein and can alter their structure upon phosphorylation; however, the mechanism by which ERK phosphorylation alters activity of the Na^+^/H^+^ exchanger still needs to be further elucidated. It is also not clear how and where other proteins bind to the intrinsically disordered region and how they alter its structure, and how this can affect function. It has been suggested that a gate exists in NHE1 that is closed upon autoinhibitory interactions with the region of the C-terminal tail [34]. However, this mechanism is based on the model of NHE1, which originated from NhaA, and others have more recently suggested that NhaA is too different from NHE1 to be used as a model [79]. Nevertheless, it seems likely that the tail must interact with the membrane domain to affect function, and regulation by phosphorylation and proteins seem likely to affect this. The structure of the intact full-length protein is highly desirable, though the intrinsically disordered domains are likely to make this difficult. A structure of a complex of NHE1 with regulatory proteins and a structure of NHE1 with induced structure in the intrinsically disordered region would be most desirable. Experiments toward this end are currently underway.

## Abbreviations

AKT	protein kinase B
CPA	Cation proton antiporter
ERK	Extracellular signal-regulated kinase
ERM	Ezrin, radixin, and moesin
MAPK	Mitogen activated protein kinase
NHE1	Na^+^/H^+^ exchanger isoform one
NMR	Nuclear magnetic resonance
pH*_i_*	Intracellular pH
